# Personalized Graphene Oxide-Protein Corona in the Human Plasma of Pancreatic Cancer Patients

**DOI:** 10.3389/fbioe.2020.00491

**Published:** 2020-05-25

**Authors:** Riccardo Di Santo, Luca Digiacomo, Erica Quagliarini, Anna Laura Capriotti, Aldo Laganà, Riccardo Zenezini Chiozzi, Damiano Caputo, Chiara Cascone, Roberto Coppola, Daniela Pozzi, Giulio Caracciolo

**Affiliations:** ^1^Nanodelivery Lab, Department of Molecular Medicine, Sapienza University of Rome, Rome, Italy; ^2^Department of Chemistry, Sapienza University of Rome, Rome, Italy; ^3^Biomolecular Mass Spectrometry and Proteomics, Bijvoet Center for Biomolecular Research and Utrecht Institute for Pharmaceutical Sciences, Utrecht University, Utrecht, Netherlands; ^4^Netherlands Proteomics Centre, Utrecht, Netherlands; ^5^General Surgery Unit, University Campus Bio-Medico di Roma, Rome, Italy

**Keywords:** protein corona, nanoparticles, graphene oxide, pancreatic ductal adenocarcinoma, precision medicine

## Abstract

The protein corona (PC) that forms around nanomaterials upon exposure to human biofluids (e.g., serum, plasma, cerebral spinal fluid etc.) is personalized, i.e., it depends on alterations of the human proteome as those occurring in several cancer types. This may relevant for early cancer detection when changes in concentration of typical biomarkers are often too low to be detected by blood tests. Among nanomaterials under development for *in vitro* diagnostic (IVD) testing, Graphene Oxide (GO) is regarded as one of the most promising ones due to its intrinsic properties and peculiar behavior in biological environments. While recent studies have explored the binding of single proteins to GO nanoflakes, unexplored variables (e.g., GO lateral size and protein concentration) leading to formation of GO-PC in human plasma (HP) have only marginally addressed so far. In this work, we studied the PC that forms around GO nanoflakes of different lateral sizes (100, 300, and 750 nm) upon exposure to HP at several dilution factors which extend over three orders of magnitude from 1 (i.e., undiluted HP) to 10^3^. HP was collected from 20 subjects, half of them being healthy donors and half of them diagnosed with pancreatic ductal adenocarcinoma (PDAC) a lethal malignancy with poor prognosis and very low 5-year survival rate after diagnosis. By dynamic light scattering (DLS), electrophoretic light scattering (ELS), sodium dodecyl sulfate-polyacrylamide gel electrophoresis (SDS-PAGE) and nano liquid chromatography tandem mass spectrometry (nano-LC MS/MS) experiments we show that the lateral size of GO has a minor impact, if any, on PC composition. On the other side, protein concentration strongly affects PC of GO nanoflakes. In particular, we were able to set dilution factor of HP in a way that maximizes the personalization of PC, i.e., the alteration in the protein profile of GO nanoflakes between cancer vs. non-cancer patients. We believe that this study shall contribute to a deeper understanding of the interactions among GO and HP, thus paving the way for the development of IVD tools to be used at every step of the patient pathway, from prognosis, screening, diagnosis to monitoring the progression of disease.

## Introduction

Upon exposure to biological milieu, nanomaterials are coated by a dynamic protein envelope, which is referred to as protein corona (PC) (Lundqvist et al., 2008; Barrán-Berdón et al., 2013). After a decade of intense research, we have clearly established that PC is shaped by several concomitant factors such as NPs’ synthetic identity, protein source and environmental factors (Pozzi et al., 2015; Ke et al., 2017). The most relevant implication is that protein patterns surrounding nanomaterials do not merely reflect the composition of the human proteome in which about twenty plasma proteins represent 99% of the total plasma volume (Schwenk et al., 2017). On the other side, PC works as “nano-concentrator” of biomolecules with an affinity for the particle surface (Zheng et al., 2015). A turning point was achieved when Mahmoudi and coworkers introduced the concept of “personalized” and “disease-specific” protein corona (Hajipour et al., 2015), i.e., they demonstrated that exposing nanomaterials to human plasma (HP) obtained from healthy subjects and patients with various diseases, induced considerable alterations in the corona profile of NPs. This is especially relevant in early detection of several cancer types where protein alteration is a hallmark of tumorigenesis and the humane proteome evolves in time as a function of the disease progress (Jimenez et al., 2018). However, no previous studies explored the concomitant effect of protein source (e.g., HP from cancer vs. non-cancer patients) and concentration on the personalization of the PC. According to previous literature (Caracciolo et al., 2011), one may expect that adjusting protein concentration by plasma dilution may modulate corona composition. For diagnostic purposes, optimal dilution factors for HP samples may defined as those boosting differences in PC composition between different classes of donors. Among technologies under development, nanoparticle-enabled blood (NEB) tests are emerging as a fast, cheap and user-friendly tool for early cancer detection (Caputo et al., 2017; Papi and Caracciolo, 2018; Caputo and Caracciolo, 2019; Papi et al., 2019). NEB tests are based on the characterization of size, surface charge and composition of the NP-PC by benchtop techniques such as dynamic light scattering (DLS), micro-electrophoresis (ME) and one-dimensional (1D) sodium dodecyl sulfate polyacrylamide gel electrophoresis (SDS-PAGE). Due to high specific surface area and the presence of carboxylic and epoxydic groups on its surface, GO exhibits high protein binding (Liu et al., 2011) through electrostatic and hydrophobic interactions (Wang et al., 2011; Chung et al., 2013). These properties makes GO an ideal candidate to differentiate blood human samples derived from different classes of individuals thus representing a logical choice for the development of new variants of the NEB test with optimized sensitivity and specificity (Lesniak et al., 2010; Castagnola et al., 2018). Recent studies have investigated molecular interactions of GO with human blood plasma proteins (Wang et al., 2018). It was demonstrated the existence of size-dependent interactions between GO nanoflakes and albumin, fibrinogen and globulin (Kenry et al., 2016). However, factors affecting adsorption from single protein solutions may be ruled out in HP by the simultaneous presence of thousands of proteins with an affinity for the particle surface. In this work we investigated the effect of GO lateral size and protein concentration on PC composition. To this end, we exposed GO nanoflakes of three different sizes (∼100, 300, and 750 nm) to diluted plasma samples with dilutions factors ranging from 1 (i.e., undiluted HP) to 10^3^. Human plasma samples were collected from 20 subjects, half of them diagnosed with pancreatic ductal adenocarcinoma (PDAC) and half of them being healthy subjects. PDAC was chosen as a model of aggressive malignancy that is predicted to become one of the leading causes of cancer-related death in the near future (Rahib et al., 2014; Caputo and Caracciolo, 2019). Surgery can increase survival rates, but regrettably, only a minority of PDACs are diagnosed early enough for resection. In the early stage the tumor is asymptomatic or symptoms can be confused with common indicators of other diseases (Caputo and Caracciolo, 2019). Therefore, the development of novel tools for the early detection of PDAC is urgently needed. Here we show that GO lateral size has no appreciable effect on PC composition. On the other hand, we demonstrate that protein concentration has a major effect on PC composition and personalization. At high protein concentration (i.e., at low plasma dilution), the PC formed in the plasma of PDAC patients was indistinguishable from that formed in the plasma of healthy subjects. On the other side, at low protein concentration (i.e., at high plasma dilution) PC of PDAC patients was significantly different from that of healthy donors. Our results suggest that concentration of plasma samples should be properly tuned to boost personalization of PC. This may be a fundamental step to improve sensitivity and sensibility of the NEB tests and other IVD technologies based on direct characterization of PC.

## Experimental Section

### Demographic Characteristics

Demographic characteristics (age, sex), tumoral markers (CEA, Ca 19.9) and gamma-globulin levels for both PDAC subjects and healthy volunteers have been evaluated (Supplementary Tables S1, S2). PDAC diagnosis was obtained in all cases with endoscopic ultrasonographic guided fine needle aspiration biopsy (EUS-FNAB). All PDAC patients were staged according to the AJCC TNM system eight edition (Chun et al., 2018). Among PDAC patients those who underwent surgery according to the good clinical practice received a pathological stage, while those who unfit for surgery received only clinical stage.

### Preparation of Graphene Oxide Nanoflakes

Graphene Oxide was purchased from Graphenea (San Sebastián, Spain). GO solutions were subjected to sonication (Vibra cell sonicator VC505, Sonics and Materials, United Kingdom) and centrifugation (Hermle Z 216 MK, Hermle Labortechnik, Germany), to obtain large, medium and small GO nanoflakes. To prepare large nanoflakes, a GO solution was subjected to a sonication of 1 min at 125 W, followed by centrifugation at 14.850 RCF for 15 min; after that the supernatant was removed, and the pellet was resuspended. To prepare medium size nanoflakes, a GO solution was sonicated (10 min, 125 W) and centrifuged two times. The first centrifuge was performed at 7.700 RCF for 30 min, then the supernatant was recovered and centrifuged again for 30 min at 18,620 RCF. Finally, the supernatant was removed, and the pellet was resuspended. Lastly, small size GO nanoflakes were obtained by sonicating three times a GO solution for 180 min at 125 W. After each sonication, the resulting solution was centrifuged at 10,000 RCF for 15 min and the final supernatant was collected. GO concentration was estimated by UV-Vis spectrometry experiments using the characteristic GO absorption peak, which is located at 230 nm (Jasim et al., 2016; Di Santo et al., 2019).

### Size and Zeta-Potential Experiments

For size and zeta-potential measurements, GO was dispersed in ultrapure water (nominal pH within 2.2–2.5). Size and zeta-potential experiments were carried out through a NanoZetaSizer apparatus (Malvern, United Kingdom) equipped with a 633 nm He–Ne laser and a digital logarithmic correlator. Size measurements were performed by using Malvern micro cuvettes (ZEN0040), while zeta-potential measurements were performed by using a Dip Cell Kit (ZEN1002). Results are reported as average ± standard deviation of three independent measurements.

### Preparation of GO-Protein Corona Complexes

Human plasma (HP) was purchased from Sigma−Aldrich, Inc. (Merk KGaA, Darmstadt, Germany). GO-protein complexes were obtained by incubating 100 μl of GO nanoflakes (0.25 mg/ml) with 100 μl of commercial HP (60 min, 37°C) at several dilution factors ranging 1–10^3^, as indicated in Supplementary Table S3. For further experiments, blood samples from 10 healthy donors and 10 PDAC patients were collected and stored according to an established protocol (10/12 ComET CBM and further amendments), which was approved by the Ethical Committee of the University Campus Bio-Medico di Roma. Blood plasma was obtained by centrifugation at 37°C for 15 min at 15,000 RCF. GO-PC complexes were obtained by incubating 100 μl of GO nanoflakes with 100 μl of blood plasma at different dilution factors, as indicated in Supplementary Table S3.

### 1D SDS PAGE Experiments

GO-PC complexes were subjected centrifugation at 18,620 RCF for 15 min at 4°C. Next, the pellet were washed with ultrapure water to eliminate free proteins (Papi et al., 2019). This procedure was repeated three time. After the washing step, the pellet was resuspended in 20 μl of Laemmli loading buffer 1×, boiled at 100°C for 10 min and centrifuged at 18,620 RCF for 15 min at 4°C. Finally, 10 μl of supernatants were recovered and loaded on a stain−free gradient polyacrylamide gel (4–20% TGX precast gels, Bio−Rad, Hercules, CA, United States) and run at 100 V for about 150 min. Gel images were obtained with a ChemiDoc^TM^ imaging system (Bio−Rad, Hercules, CA, United States) and were processed by ImageLab Software and custom Matlab (MathWorks, Natick, MA, United States) scripts to evaluate the one-dimensional intensity distribution function of each sample and obtain the corresponding one-dimensional molecular weight (MW) distribution. On each of the gel images, background was preliminary removed to prevent unwanted baselines affect the resulting curves. In detail, a rolling ball subtraction was carried out, row by row with a fixed rolling ball radius. Sampled intensity *I*(*x*,*y*) of the original image was therefore converted into a new two-variable function *I*’(*x*,*y*):

$$I\,\left(x,y\right)\to I^{\prime }\,\left(x,y\right)$$

where the *y*-displacement is related to protein MWs, x-displacement is a discrete sample index and the intensity value is proportional to the detected protein amount. The projection (*P*) of the two-variable function over a plane orthogonal to the image is the resulting protein pattern of the generic lane (*j*):

$$P_{j}\,\left(y\right)=I^{\prime }\,\left(x_{j},y\right)$$

Then, *y*-displacements were converted into MW values, by fitting the location of known proteins (ladder lane) to the following non-linear equation:

$$M\,W\,\left(y\right)=a_{1}\,e^{b_{1}\,y}+a_{2}\,e^{b_{2}\,y}$$

The resulting one-dimensional profiles represent the MW distributions of the corresponding samples and were finally normalized to 1. Normalized distributions were subdivided into MW ranges, within which the corresponding definite integrals were computed. Further details can be found elsewhere (Digiacomo et al., 2019).

### Nano Liquid Chromatography Tandem Mass Spectrometry

GO nanoflakes were incubated with HP (1:200 vol/vol) for 1 h at 37°C. Then, samples were centrifuged three times for 15 min at 18,620 RCF at 4°C. Pellets were vigorously washed, each time, with ultrapure water to remove loosely bound proteins. After washes, pellets were subjected to protein denaturation, digestion, and desalting following a robust protocol which is generally applied to isolate unbound and loosely bound proteins from bio-coronated materials (Chetwynd et al., 2019). Finally, the samples were lyophilized using a Speed-Vac device (mod. SC 250 Express; Thermo Savant, Holbrook, NY, United States), reconstituted with 0.1% HCOOH solution and stored at −80°C until use. Tryptic peptides were investigated by using a nano-liquid chromatography apparatus (Dionex Ultimate 3000, Sunnyvale, CA, United States) connected to a hybrid mass spectrometer (Thermo Fisher Scientific, Bremen, Germany) and equipped with a nanoelectrospray ion source. Xcalibur (v.2.07, Thermo Fisher Scientific) raw data files were submitted to Proteome Discover (1.2 version, Thermo Scientific) for a database search using Mascot (version 2.3.2 Matrix Science). Data were searched against the SwissProt database (v 57.15, 20 266 sequences) using the decoy search option of Mascot and protein quantification was performed using Scaffold software. For each identified protein, the mean value of the normalized spectral countings (NSCs) was normalized to the protein molecular weight (MWNSC) to obtain the relative protein abundance (RPA). For each identified protein, the reported RPA is the mean of three independent technical replicates ± standard deviation.

## Results

### Effects of GO Lateral Size on PC Composition

**FIGURE 1:**
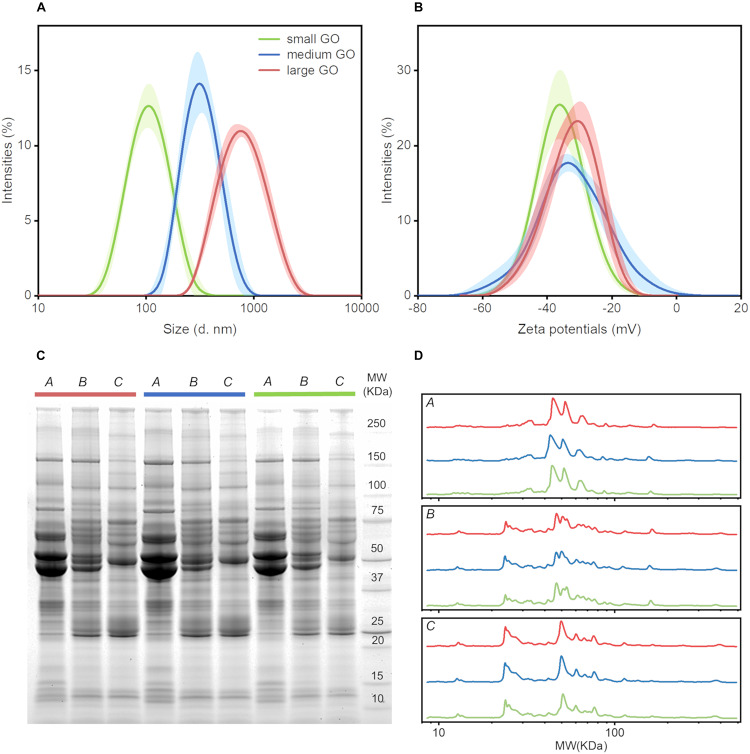
Characterization of GO nanoflakes and their interaction with human plasma (HP). **(A)** Size and **(B)** zeta-potential distributions of small, medium and large nanoflakes. **(C)** Representative SDS-PAGE image of PC on small, medium and large GO nanoflakes upon incubation with HP at three different dilution factors: *A* = 1 (i.e., undiluted HP), *B* = 5 and *C* = 10, respectively. The normalized intensity profile of each lane is reported in **(D)**.

Our first aim was to investigate the effect of GO lateral size on PC composition. To this end, we used small, medium and large GO nanoflakes with size distribution centered at ∼100, 300, and 750 nm respectively (Figure 1A). To explore whether surface charge of GO nanoflakes was influenced by lateral size, zeta-potential experiments were performed. It was interesting to observe that no variation in surface charge was caused by changing the lateral size. In fact, zeta-potential distributions of small, medium and large GO nanoflakes were comparable and peaked around −30 mV (Figure 1B). Next, we evaluated whether the molecular interactions between GO and blood plasma proteins were affected by GO lateral size. To this end, GO nanoflakes were incubated with HP at different dilution factors. SDS-PAGE results (Figure 1C) indicate that PC formed around GO nanoflakes was affected by protein concentration, but not by GO lateral size. In Figure 1D we report normalized MW distributions of protein patterns. The three boxes regroup the intensity profiles for each plasma dilution at the three different sizes of GO nanoflakes. As expected by the visual inspection of the gel image, profiles trends are almost superimposable for small, medium and large GO-protein complexes but strongly distinct for dilution conditions (dilution factors *A* = 1, *B* = 5, *C* = 10 respectively). Thus, we demonstrated that the molecular interactions between GO and proteins are strongly influenced by protein concentration and not influenced by the GO lateral size. As a consequence, in the following experiments only large size GO nanoflakes were used.

### Effects of Protein Concentration on Protein Corona Composition

**FIGURE 2:**
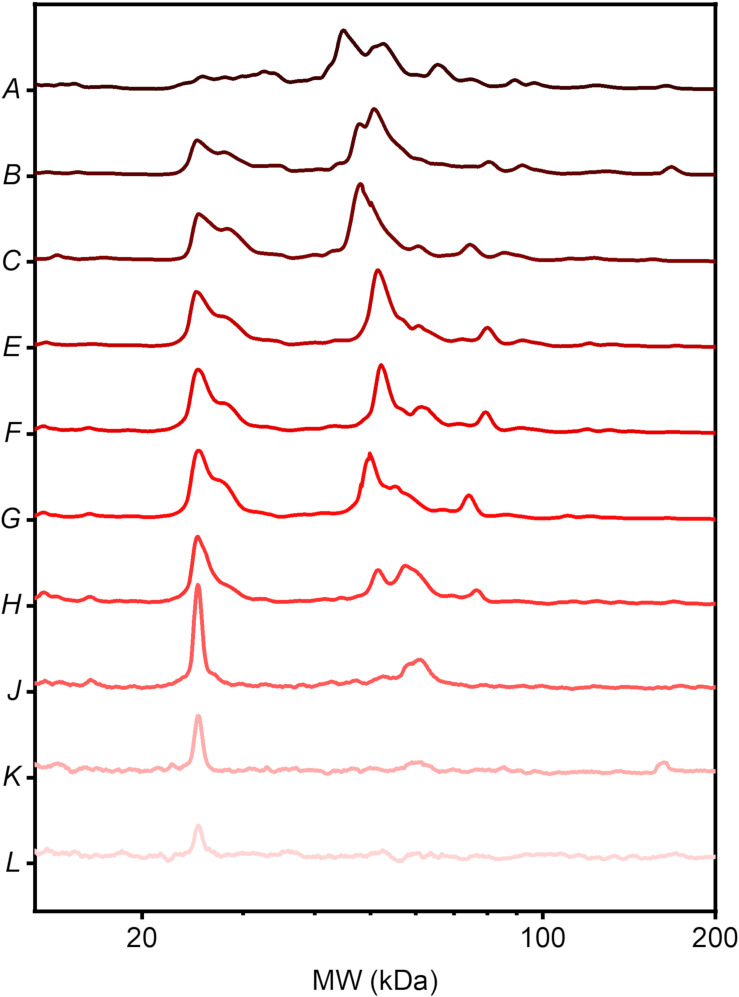
SDS-page profile analysis of coronated GO nanoflakes upon incubation with HP at increasing dilution factors ranging from *A* = 1 to *L* = 10^3^.

Motivated by results of Figure 1 we characterized PC of GO nanoflakes in a much wider protein concentration range. To accomplish this issue, GO nanoflakes were incubated with HP at 12 dilution factors ranging from dilution factor *A* = 1 (i.e., undiluted HP) to dilution factor *L* = 10^3^. Intensity profiles corresponding to each dilution factor are reported in Figure 2. At the lowest protein concentration (i.e., at the highest dilution factor, *L* = 10^3^) the intensity profile exhibited a single evident peak around 25 kDa. On the other side, the complexity of the protein pattern increased with increasing protein concentration. Furthermore, Figure 2 also shows that MW distribution of corona proteins is markedly shaped by plasma dilution. This finding may have relevant implications for cancer detection. Indeed, it is a hallmark of tumorigenesis that cancer induces relevant changes in concentration of several blood plasma proteins with different MW (Shruthi and Palani Vinodhkumar, 2016). The situation is even more complex if one considers that these alterations may be also influenced by cancer stage and clinical treatment. As a consequence, one may predict that only exposing nanomaterials to HP at certain plasma dilution factors may promote enrichment of plasma proteins whose blood concentration has been altered by cancer onset and progression. To explore this further, we exposed GO nanoflakes to plasma samples from PDAC patients and healthy subjects under different dilution conditions and compared the emerging coronas.

### Personalized Protein Corona in PDAC Patients

**FIGURE 3:**
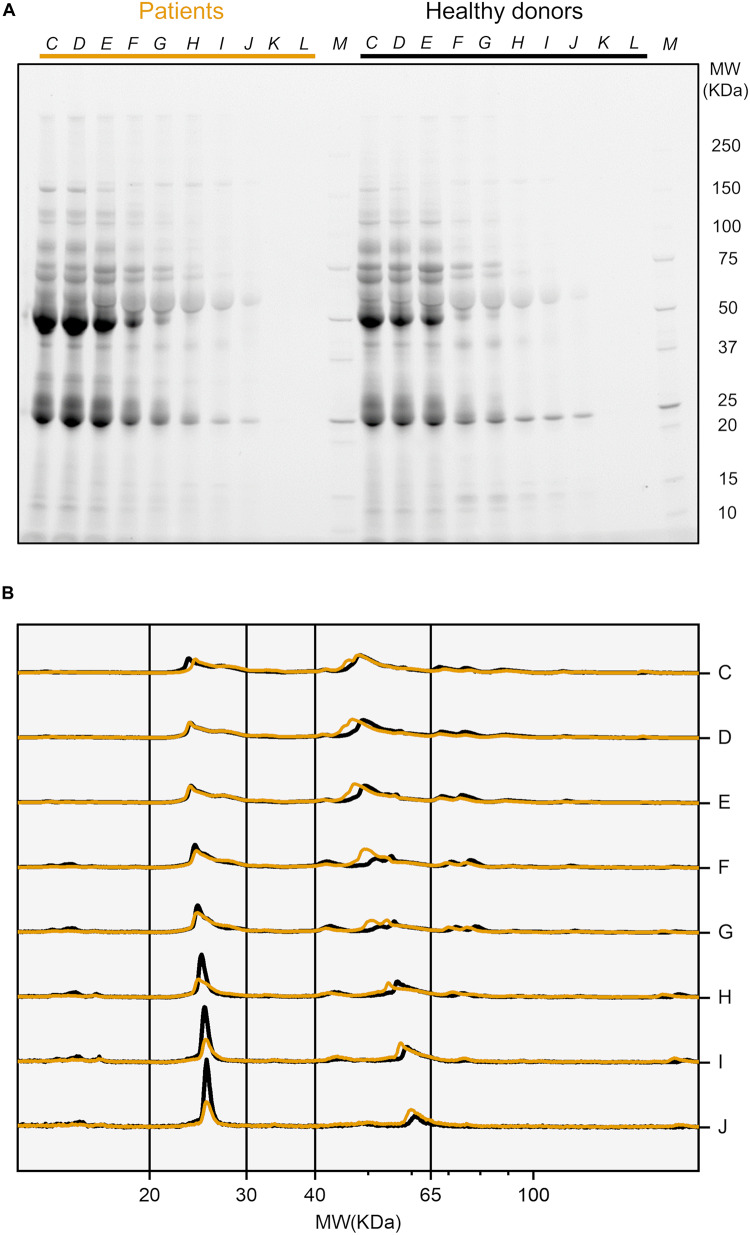
**(A)** Representative SDS-page image of biocoronated GO nanoflakes upon incubation with human plasma from PDAC patients and healthy volunteers. Plasma samples were diluted with dilution factors ranging from *C* = 10 to *L* = 10^3^. **(B)** One-dimensional protein profiles obtained by densitometry analysis of SDS-PAGE results. Profiles K and L are not reported because their low signal-to-noise ratio compromised the analysis.

**FIGURE 4:**
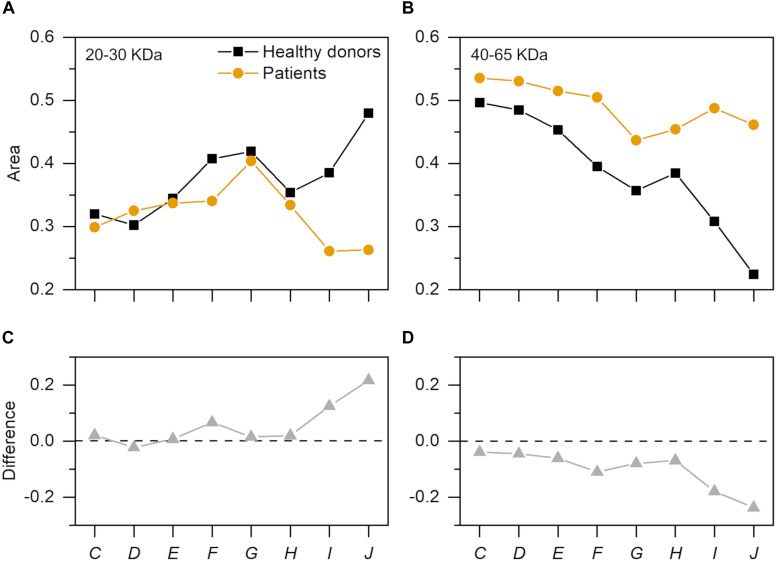
Integral analysis of electrophoretic profiles. Integral area of SDS-PAGE intensity profiles within **(A)** 20–30 kDa and **(B)** 40–65 kDa. Differences among healthy donors and PDAC patients is reported in **(C)** and **(D)**.

GO nanoflakes were exposed to diluted plasma samples from 10 healthy HP donors and 10 PDAC patients. A representative gel image is presented in Figure 3A. For better comparison, we also performed a densitometric analysis of each gel lane. In Figure 3B we compare the intensity profiles derived from PDAC patients and healthy donors (yellow and black curves respectively). Vertical solid lines indicate two MW ranges where protein profiles were differentiated. Between 20 and 30 kDa a sharp peak is present in healthy profiles, while it is definitely less pronounced in PDAC patients. In the second MW range (from 40 to 65 kDa), slighter but significant variations were detected. To compare coronas of PDAC patients and healthy volunteers, we calculated the integral areas of the experimental distributions within the two selected MW ranges. Figure 4A shows that the gap between the integral area of healthy donors and PDAC patients in the first MW range (20–30 kDa) increases with increasing plasma dilution. An opposite trend was found in the second MW range (40–65 kDa) (Figure 4B). Then, we calculated the relative differences by subtracting the areas derived from healthy donors from those derived from PDAC patients (Figures 4C,D). Comparing results of Figures 4C,D one may speculate that at high dilution (dilution factor *J* = 200) PDAC induces reduction in relative abundance of plasma proteins with MW between 20 and 30 kDa that is compensated by simultaneous enrichment of proteins with MW comprised between 40 and 65 kDa. In conclusion, SDS-PAGE results indicate that PDAC can induce personalization of the PC that forms around GO nanoflakes in HP. Most importantly, the occurrence and relevance of this process is markedly influenced by protein concentration.

### Liquid Chromatography Tandem Mass Spectrometry

**FIGURE 5:**
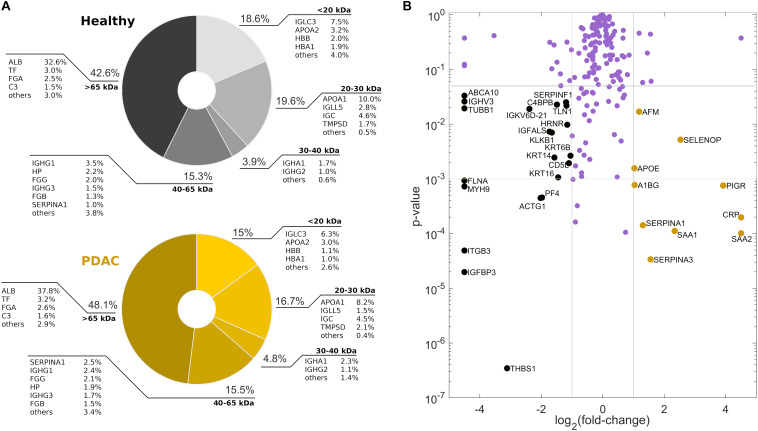
Protein corona composition by mass spectrometry experiments. **(A)** Pie charts and **(B)** volcano plot of the detected proteins in the corona of healthy donors and PDAC patients. Horizontal thresholds in **(B)** are set as *p* < 0.05 and *p* < 0.001, vertical thresholds are set at −1 and 1.

As a next step, we performed nano-LC MS/MS experiments to identify and quantify proteins enriching the coronas of GO nanoflakes following exposure to plasma samples of 10 PDAC patients and 10 healthy volunteers. According to SDS-PAGE results, dilution factor 200 (condition J) was chosen as it boosts differences in PC composition. 199 proteins were detected on the studied samples (Supplementary Table S4), but only 20 of them represented the most abundant parts of the coronas, with a cumulative relative protein abundance (RPA) that reached about 88 and 90%, for healthy and PDAC samples respectively. Those 20 proteins were the only ones with RPAs larger than 1%. Their gene names are reported around the pie charts in Figure 5A, which shows the measured distributions of the corona proteins according to their MW. Interestingly, healthy PCs were more enriched by low-MW proteins than PDAC counterparts. In detail, differences of about 3% were detected within the first two MW ranges (i.e., <20 and 20–30 kDa). Apolipoprotein A1 was one of the most abundant proteins (second largest RPA, after human serum albumin) and exhibited a manifest decrease in PDAC samples. On the other hand, alpha-1-antitrypsin (serpina1) and serum albumin were more abundant in PDAC coronas with respect to the healthy ones. Indeed, the former was 2.5 times more enriched for PDAC and the latter exhibited the highest absolute difference (about 5%). Finally, to take into account proteins that may show significant differences despite their small RPAs, we compared the PDAC-to-healthy fold-change of the measured RPAs and the corresponding *p*-value from Student’s *t* test, for each of the detected corona protein. Data are shown in Figure 5B, as a Volcano plot. Each protein is represented by a dot, whose location is determined by the abundance’s excess in healthy (left region) or PDAC (right region) coronas and the corresponding statistical significance (with the commonly employed thresholds *p* < 0.05 and *p* < 0.001). Black and yellow dots correspond to potential biomarkers for PDAC as they indicate proteins that were found to be strongly downregulated (black) or upregulated (yellow) in healthy and cancer corona respectively.

## Discussion

Physical-chemical properties of pristine nanomaterials are the main factors driving the interactions of NPs in biological environments. In fact, size, curvature radius, surface charge, surface chemistry and functionalization represent some of the interdependent features that co-determine NP behavior in biological media, as those properties affect protein adsorption on NP surface, thus formation and composition of the resulting PC and the subsequent physiological response. This could be important for early detection of several human conditions that cause alteration in the proteome. A paradigmatic example is provided by cancer where protein alteration is frequently associated to the disease onset and progression. Moreover, recent research has suggested that systematic comparisons of corona patterns of nanoparticles recovered from cancer patients and healthy individuals may help to discover new insights on the biology and stage of distinct types of cancer (Caputo et al., 2018; Caracciolo et al., 2019). Nanomaterials coated by a disease-specific PC could be therefore designed to develop cancer detection technologies aligned to the ASSURED criteria stated by the World Health Organization for cancer screening and detection. More in depth investigations on larger cohorts are needed to validate clinical applicability of PC-based detection technologies, and to evaluate the factors that may influence their specificity and sensitivity. This study was aimed at providing new insights on the role of GO lateral size and plasma protein concentration on the personalization of the PC. For two-dimensional materials, previous studies explored size effects on the interactions of the designed NPs with single plasma proteins (Kenry et al., 2016) and living systems (Ma et al., 2015). Here, we firstly studied the role of GO lateral size on the composition of the PC that was formed upon exposure to HP. GO solutions were obtained by an iterative sonication/centrifugation procedure, therefore surface chemistry and oxidation state were maintained the same for all the resulting samples, which differed only for their lateral size. Figures 1A,B clearly show that we were able to separate the starting batch of GO solution into three distinct populations with different lateral size and almost equal zeta potential. Then, after exposure to human plasma (HP), we evaluated the protein patterns of the PC formed on small, medium and large GO sheets by 1D SDS-PAGE experiments. Interestingly, we found that GO lateral size have minor impact, if any, on PC composition, whereas protein concentration strongly affected the measured protein patterns (Figures 1C,D). This outcome was relatively surprising, as it seemed to be in disagreement with previous findings. As an instance, Kenry et al. studied adsorption, binding kinetics, conformational stability and structural change of single proteins interacting with GO and concluded that molecular interactions between GO and plasma proteins are significantly dependent on the lateral size distribution of GO sheets (Kenry et al., 2016). Our results suggest that despite the molecular interactions of single proteins may be affected by GO lateral size, those size effects become negligible in physiological environments, where single GO-protein binding parameters represent only a portion of a greater description, which involves protein-protein interactions and competitive adsorption mechanisms. Our evidence shows that in a complex and crowded protein source such HP, the overall composition of the PC does not depend on GO lateral size, at least within the explored size range. Furthermore, even for the smallest GO sheets population (lateral size ∼100 nm), the available surface area for protein adsorption is much greater than the average cross section of any single protein. Indeed, under both rough and more detailed approximations, the Stokes radius of proteins (modeled as spherical objects) (Erickson, 2009) is on average more than one order of magnitude smaller than the smallest GO population employed in this work. Therefore, it is reasonable suppose that possible size dependence of protein adsorption, if any, may be due to edge effects that become negligible as GO lateral size increases. On the other hand, protein concentration is a determining factor involved in formation and shaping of the PC on manifold nanomaterials (Lesniak et al., 2010; Caracciolo et al., 2011; Castagnola et al., 2018) and this trend has been already confirmed for graphene-based systems. As an instance, Castagnola et al. founded that the electrophoretic protein profile of Graphene-PC complexes was not modified only above a certain threshold of protein concentration (50% v/v) (Castagnola et al., 2018). In agreement with that work, our findings extend the explored incubation conditions, spanning a very broad range of plasma concentration (Figure 2). Interestingly, the obtained electrophoretic patterns exhibited clear trends, especially within 20–30 and 40–65 kDa (Figures 3, 4). To further clarify SDS-PAGE results, we identified proteins enriching PC by nanoLC MS/MS. In particular, within 20–30 kDa proteins apolipoprotein A1 (APOA1) was found to be the most enriched plasma protein (Figure 5A). By recent experimental evidence, it is known that apolipoprotein A1 has a high affinity to graphene and GO surface (Castagnola et al., 2018; Alnasser et al., 2019), and, interestingly, that protein is recognized as a potential biomarker for PDAC (Koomen et al., 2005; Brand et al., 2011; Lin et al., 2016). Within the other MW ranges, some representative proteins are serum albumin (ALB), hemoglobin (subunit B and A1, HBB and HBA1 respectively) and alpha-1-antitrypsin (SERPINA1). In previous studies, SERPINA1 was identified as a clinically useful biomarker for prognostic or therapeutic purposes in metastatic pancreatic cancer (Koomen et al., 2005; Thakur et al., 2008). Interestingly, in our analysis SERPINA1 was among those corona proteins that had a significant PDAC-to-healthy RPA fold-change, despite their low abundance (Figure 5B). This particular group includes also serum amyloid proteins (SAA1 and SAA2), apolipoprotein E (APOE) and alpha 1B glycoprotein (A1BG). SAA is an acute-phase protein involved in tumor pathogenesis and might be included in a group of non-specific biomarkers for cancers, including PDAC (Malle et al., 2009). APOE has been found to be up-regulated in the sera of patients with PDAC and has been proposed as a potential PDAC-related biomarker, alone or in combination with others (Chen et al., 2013; Lin et al., 2016). Finally, A1BG is a plasma glycoprotein, whose function is not clear yet but its overexpression has been detected and quantified in cancerous pancreatic juice (Tian et al., 2008). These are some representative examples of known potential biomarkers for cancers that were detected by GO protein corona. In principle, the list could be enlarged with other proteins, which exhibit variations in the personalized coronas but to date are not yet known to have a role in the tumor biology of PDAC.

## Conclusion

**FIGURE 6:**
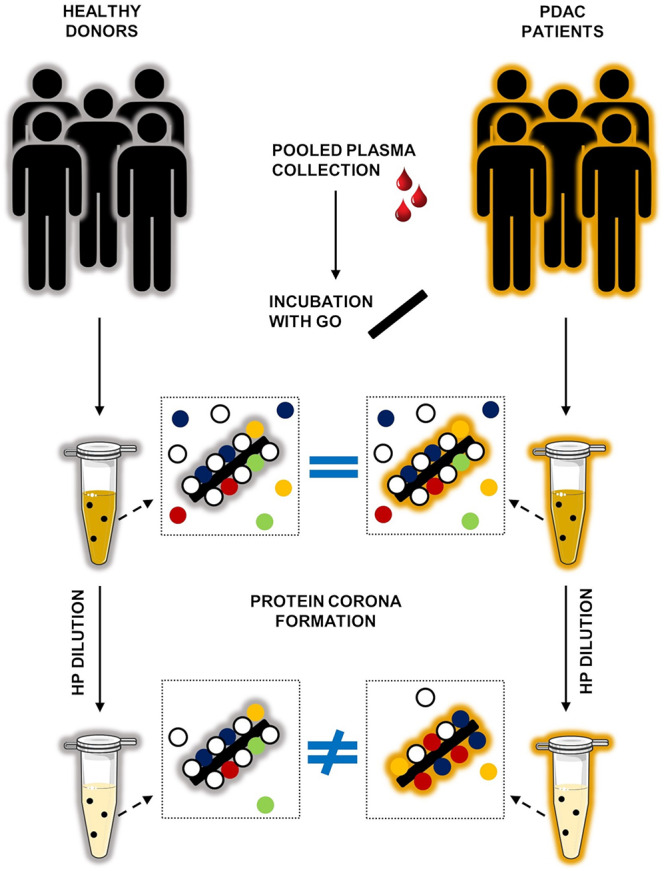
Personalization of protein corona depends on protein concentration. Cartoon describing our present understanding of the role of protein concentration on personalization of protein corona. When GO nanoflakes are incubated with human plasma (HP) a complex protein corona forms at the GO surface. When undiluted HP is used, the coronas of healthy donors and PDAC patients are not discernible. On the other side, after exposure of GO nanoflakes to highly diluted HP (dilution factor >200) characterization of the protein corona allows to distinguish between the two classes of donors.

In conclusion, we have shown that the protein corona that forms around GO nanoflakes in human plasma does not depend on GO lateral size but is strongly affected by protein concentration. The latter result may be relevant to exploit the personalized protein corona for diagnostic purposes. Indeed, we could demonstrate that the protein corona of pancreatic cancer patients differs significantly from that of healthy individuals only at very low protein concentration, i.e., upon 200-fold dilution of human plasma. The main limitation of the present study is undoubtedly the use of pooled samples. Although employment of non-pooled samples is a key issue for the validation of PC-based diagnostic tests, this is far beyond the scope of the present investigation. We indeed see this work as the proof of concept that exposing nanomaterials to plasma samples under ‘optimal dilution conditions’ could be a good strategy to amplify personalization of the protein corona and, in turn, to exploit it to distinguish between different classes of donors (e.g., cancer vs. non-cancer patients) (Figure 6). We underline that identification of optimal dilution factors may be not trivial as it may depend simultaneously on synthetic identity of nanomaterials, the protein source and environmental factors. We envision that exploring the interactions among nanomaterials and HP will offer unprecedented opportunities for the development of sophisticated IVD technologies to be used at every step of the patient pathway, from diagnosis to monitoring the progression of disease and individual response to treatment.

## Data Availability Statement

The datasets generated for this study are available on request to the corresponding author.

## Ethics Statement

The studies involving human participants were reviewed and approved by Ethics Committee of the University Campus Bio-Medico di Roma. The patients/participants provided their written informed consent to participate in this study.

## Author Contributions

DC, RC, AL, GC, and DP: conceptualization. GC and DP: methodology, project administration, and funding acquisition. LD: software and data curation. RD and LD: formal analysis, writing – original draft preparation, and visualization. RD, LD, EQ, CC, and AC: investigation. DC, RZ, GC, and DP: resources and writing – review and editing. AL, GC, and DP: supervision.

## Conflict of Interest

The authors declare that the research was conducted in the absence of any commercial or financial relationships that could be construed as a potential conflict of interest.
