# Supplementary material for: Personalized Graphene Oxide-Protein Corona in the Human Plasma of Pancreatic Cancer Patients
Source: Front Bioeng Biotechnol. 2020 May 25;8:491. doi: 10.3389/fbioe.2020.00491 (PMC7261887; doi:10.3389/fbioe.2020.00491)
Supplement: Supplementary file 1 [file Data_Sheet_1.PDF]

Supplementary Material

Personalized graphene oxide-protein corona in the human plasma of pancreatic cancer patients

Riccardo Di Santo[1#], Luca Digiacomo[1#], Erica Quagliarini[2], Anna Laura Capriotti[2], Aldo Laganà[2], Riccardo Zenezini Chiozzi[3,4], Damiano Caputo,[5] Chiara Cascone,[5] Roberto Coppola,[5] Daniela Pozzi[1*], Giulio Caracciolo[1*]

[1]Nanodelivery Lab, Department of Molecular Medicine, Sapienza University of Rome, Rome, Italy

[2]Department of Chemistry, Sapienza University of Rome, Rome, Italy

[3]Biomolecular Mass Spectrometry and Proteomics, Bijvoet Center for Biomolecular Research and Utrecht Institute for Pharmaceutical Sciences, Utrecht University, Utrecht, The Netherlands

[4]Netherlands Proteomics Centre, Utrecht, The Netherlands

[5]General Surgery, University Campus Bio-Medico di Roma, Rome, Italy

[#] equal contribution

*** Correspondence:**
Corresponding Author: giulio.caracciolo@uniroma1.it; daniela.pozzi@uniroma1.it

Keywords: Protein Corona; Nanoparticles; Graphene Oxide; Pancreatic Ductal Adenocarcinoma

Table S1. Demographic and clinical characteristics of PDAC and control group.

	PDAC patients (N=10)	Healthy subjects (N=10)
**Age, years**		
Median (range)	73 (54 - 80)	57.5 (27 - 78)
**Sex n (%)**		
Male, n (%)	6 (60%)	4 (40%)
Female, n (%)	4 (40%)	6 (60%)
**Diagnosis n (%)**		
Surgery	8 (80%)	NA
Biopsy	2 (20%)	NA
**CEA (n.v. < 5 ng/mL)**		
< 5 ng/mL	8 (80%)	10 (100%)
> 5 ng/mL	2 (20%)	0 (0%)
**CA 19.9 (n.v. < 37 UI/mL)**		
< 37 UI/mL	2 (20%)	10 (100%)
> 37 UI/mL	8 (80%)	0 (0%)
**TNM stage (AJCC 8th edition) n**		
**(%)**	-	-
IA	-	-
IB	-	-
IIA	9 (90%)	-
IIB	-	-
III	1 (10%)	-
IV		

(n.v. normal value; NA not applicable).

Table S2. Gamma globulin levels detected by electrophoretic analysis in PDAC and control group.

**Gamma Globulin, %<br>(n.v. 11.10 - 18.80)**	
**PDAC patients (N=10)**	**Healthy subjects (N=10)**
19.5	12.9
15.3	16.4
13.7	10.6
13	12.3
15.8	18.6
12.1	13.8
13.5	23.4
16.2	18.3
15.2	17.1
15.2	10.2

n.v. normal value

Table S3. List of samples and corresponding dilution factors of human plasma.

**Sample name**	**Dilution factor**
A	1
B	5
C	10
D	13
E	20
F	40
G	50
H	100
I	133
J	200
K	400
L	1000

Table S4. Human plasma proteins identified in the coronas of GO nanoflakes following 1-hour exposure to HP of 10 healthy donors and 10 patients diagnosed with pancreatic ductal adenocarcinoma (PDAC). For each identified protein, the reported RPA is the mean of three independent technical replicates ± standard deviation (Error).

#	Gene name	Mol. weight [kDa]	Isoelectric point	RPA Healthy	Error	RPA PDAC	Error
1	TMSB4X	5,0526	4,884	0,005%	0,009%	0,000%	0,000%
2	APOC1	9,3318	8,757	0,327%	0,044%	0,217%	0,014%
3	PF4;PF4V1	10,845	8,877	0,072%	0,008%	0,018%	0,004%
4	APOC3	10,852	5,121	0,191%	0,095%	0,129%	0,070%
5	APOA2	11,175	6,438	3,178%	0,292%	2,996%	0,526%
6	IGLC7	11,253	8,19	0,135%	0,007%	0,076%	0,006%
7	IGLC3;IGLC2	11,265	6,967	7,462%	1,301%	6,307%	0,726%
8	APOC2	11,284	4,499	0,202%	0,027%	0,315%	0,065%
9	DCD	11,284	6,227	0,112%	0,021%	0,106%	0,014%
10	IGKC	11,765	5,599	0,678%	0,152%	0,607%	0,268%
11	IGLV2-23	11,893	8,585	0,009%	0,016%	0,001%	0,001%
12	IGLV3-25;IGLV3-16;IGLV3-27	12,011	4,468	0,063%	0,011%	0,075%	0,013%
13	IGLV3-19	12,042	6,187	0,076%	0,007%	0,061%	0,005%
14	IGLV1-51	12,249	7,897	0,110%	0,060%	0,088%	0,055%
15	IGLV1-47;IGLV1-44	12,283	6,946	0,092%	0,008%	0,074%	0,002%
16	IGLV1-40	12,301	9,457	0,023%	0,002%	0,014%	0,001%
17	IGLV3-9;IGLV3-21;IGLV3-12	12,332	5,357	0,073%	0,011%	0,052%	0,010%
18	IGKV6D-21	12,34	7,041	0,010%	0,002%	0,002%	0,003%
19	IGLV10-54	12,395	7,927	0,013%	0,012%	0,000%	0,000%
20	IGKV6-21	12,43	7,041	0,007%	0,006%	0,000%	0,000%
21	IGLV3-10	12,441	4,7635	0,234%	0,032%	0,280%	0,059%
22	IGLV7-46;IGLV7-43	12,468	6,834	0,054%	0,012%	0,073%	0,007%
23	IGKV3-15;IGKV3D-7	12,496	7,3325	0,163%	0,030%	0,169%	0,032%
24	IGHV3-13;IGHV3-20;IGHV3-43D;IGHV3-43;IGHV3-9	12,506	6,69	0,000%	0,000%	0,001%	0,002%
25	IGKV3D-20	12,515	4,306	0,004%	0,007%	0,011%	0,004%
26	IGKV3D-15	12,534	5,007	0,006%	0,001%	0,006%	0,001%
27	IGKV3-20	12,557	9,367	0,118%	0,021%	0,100%	0,005%
28	IGKV3D-11;IGKV3-11	12,625	4,875	0,007%	0,002%	0,021%	0,027%
29	IGHV1-69D;IGHV1-8;IGHV1-3;IGHV1-46;IGHV1-69	12,66	8,7265	0,015%	0,005%	0,020%	0,003%
30	IGHV5-51	12,674	8,167	0,022%	0,004%	0,022%	0,006%
31	IGLV4-69	12,773	6,173	0,027%	0,025%	0,030%	0,004%
32	IGKV1-17;IGKV1-6	12,778	8,585	0,009%	0,002%	0,014%	0,009%
33	IGHV3-64D	12,822	7,704	0,025%	0,011%	0,043%	0,008%
34	IGKV1-33;IGKV1D-33	12,848	5,326	0,010%	0,003%	0,008%	0,000%
35	IGHV1-69-2	12,87	4,58	0,000%	0,000%	0,000%	0,000%
36	IGHV3-64	12,891	7,709	0,005%	0,005%	0,005%	0,004%
37	IGHV3-15;IGHV3-73	12,926	8,9615	0,039%	0,007%	0,023%	0,005%
38	IGHV3-7;IGHV3-21;IGHV3-48;IGHV3-11	12,943	8,968	0,174%	0,037%	0,148%	0,016%
39	IGHV3-30-5;IGHV3-30;IGHV3-23	12,947	8,9625	0,007%	0,004%	0,000%	0,000%
40	IGKV2-28;IGKV2-40;IGKV2D-28;IGKV2D-40;IGKV2D-30	12,957	5,621	0,252%	0,044%	0,201%	0,022%
41	IGLV4-60	12,987	5,933	0,004%	0,008%	0,000%	0,000%
42	IGHV3-30-3;IGHV3-33;IGHV3-66;IGHV3-53	12,989	8,017	0,192%	0,061%	0,160%	0,018%
43	IGHV3-49	13,056	8,594	0,004%	0,001%	0,003%	0,001%
44	IGHV4-28	13,124	9,43	0,004%	0,007%	0,005%	0,008%
45	IGKV2-30	13,185	9,388	0,006%	0,005%	0,002%	0,002%
46	IGHV3-72	13,203	7,71	0,002%	0,002%	0,006%	0,001%
47	IGKV4-1	13,38	4,945	0,039%	0,003%	0,041%	0,008%
48	IGLV5-39;IGLV5-45	13,394	7,7315	0,023%	0,006%	0,014%	0,009%
49	IGHV6-1	13,481	9,337	0,015%	0,001%	0,013%	0,005%
50	SAA2	13,527	9,308	0,000%	0,000%	0,004%	0,000%
51	SAA1	13,532	6,481	0,015%	0,001%	0,074%	0,007%
52	IGHV4-34;IGHV4-38-2;IGHV4-30-4;IGHV4-61;IGHV4-59;IGHV4-39	13,815	8,93	0,047%	0,033%	0,021%	0,020%
53	PPBP	13,894	8,799	0,023%	0,013%	0,007%	0,002%
54	APOC4	14,553	8,885	0,000%	0,000%	0,002%	0,003%
55	SAA4	14,746	9,206	0,082%	0,033%	0,068%	0,020%
56	HBA1	15,257	8,555	1,938%	0,284%	0,974%	0,136%
57	CST3	15,799	8,72	0,002%	0,002%	0,001%	0,001%
58	TTR	15,887	5,5	0,120%	0,015%	0,100%	0,010%
59	HBB	15,998	6,956	2,017%	0,204%	1,091%	0,069%
60	HBD	16,055	7,403	0,010%	0,001%	0,009%	0,002%
61	LYZ	16,537	9,165	0,005%	0,004%	0,005%	0,004%
62	PRH1	17,016	4,49	0,005%	0,005%	0,005%	0,001%
63	JCHAIN	18,098	4,964	0,047%	0,003%	0,038%	0,009%
64	RAP1A;RAP1B	20,987	5,513	0,000%	0,000%	0,000%	0,000%
65	APOM	21,253	5,729	0,024%	0,007%	0,024%	0,007%
66	APOD	21,275	4,932	0,002%	0,001%	0,002%	0,001%
67	C8G	22,277	8,24	0,056%	0,005%	0,048%	0,003%
68	CLEC3B	22,537	5,44	0,014%	0,006%	0,005%	0,001%
69	IGLL5;IGLC1	22,83	8,2545	2,831%	0,156%	1,528%	0,053%
70	RBP4	23,01	5,749	0,077%	0,015%	0,062%	0,008%

71	IGC	23,379	8,163	4,636%	0,473%	4,477%	0,332%
72	ORM1	23,511	4,803	0,001%	0,001%	0,003%	0,001%
73	TMPRSS13	24,409	8,457	1,742%	0,234%	2,210%	0,206%
74	CRP	25,038	5,386	0,000%	0,000%	0,015%	0,002%
75	GPX3	25,552	8,014	0,003%	0,001%	0,003%	0,002%
76	C1QC	25,773	8,318	0,060%	0,009%	0,048%	0,007%
77	C1QA	26,016	9,236	0,014%	0,004%	0,010%	0,001%
78	C1QB	26,721	8,609	0,033%	0,003%	0,034%	0,003%
79	CFD	27,033	7,488	0,002%	0,001%	0,001%	0,002%
80	TUBA4B;TUBA4A;TUBA8;TUBA1C;TUBA1B	27,551	4,821	0,000%	0,000%	0,000%	0,000%
81	C4BPB	28,357	4,933	0,026%	0,007%	0,009%	0,004%
82	CA1	28,87	6,804	0,013%	0,008%	0,005%	0,002%
83	CFHR2	30,65	6,059	0,002%	0,002%	0,002%	0,001%
84	APOA1	30,777	5,524	10,043%	1,214%	8,184%	0,805%
85	IGFBP3	31,674	8,523	0,001%	0,000%	0,000%	0,000%
86	FCN3	32,903	6,338	0,017%	0,001%	0,022%	0,001%
87	IGHG2	35,9	7,38	1,033%	0,129%	1,088%	0,138%
88	IGHG4	35,94	7,095	0,459%	0,103%	0,361%	0,032%
89	APOE	36,154	5,544	0,197%	0,025%	0,403%	0,039%
90	CFHR1	37,65	7,146	0,011%	0,002%	0,010%	0,002%
91	IGHA1	37,654	6,172	1,675%	0,105%	2,319%	0,080%
92	CD5L	38,087	5,203	0,027%	0,001%	0,013%	0,003%
93	APOH	38,298	7,777	0,164%	0,021%	0,115%	0,009%
94	LUM	38,429	6,294	0,001%	0,000%	0,002%	0,001%
95	AMBP	38,999	5,959	0,009%	0,009%	0,006%	0,001%
96	HPR	39,029	6,76	0,031%	0,010%	0,021%	0,008%
97	AHSG	39,34	5,437	0,266%	0,057%	0,389%	0,045%
98	TSPY26P	39,572	7,943	0,007%	0,006%	0,009%	0,002%
99	PON1	39,731	4,988	0,012%	0,007%	0,010%	0,002%
100	IGHG3	41,287	7,711	1,496%	0,062%	1,707%	0,086%
101	ACTG1;ACTB	41,792	5,908	0,071%	0,009%	0,017%	0,002%
102	SELENOP	43,173	7,68	0,007%	0,002%	0,041%	0,010%
103	APOL1	43,974	5,565	0,022%	0,004%	0,015%	0,005%
104	HP	45,205	6,253	2,233%	0,100%	1,940%	0,101%
105	APOA4	45,398	5,178	0,273%	0,046%	0,202%	0,028%
106	SERPINA5	45,674	9,469	0,002%	0,001%	0,001%	0,000%
107	SERPINF1	46,312	6,083	0,014%	0,003%	0,006%	0,002%
108	LRR1	46,722	9,18	0,015%	0,003%	0,008%	0,004%
109	SERPINA1	46,736	6,544	1,024%	0,122%	2,531%	0,138%
110	SERPINA3	47,65	5,255	0,049%	0,004%	0,144%	0,007%
111	SERPINA4	48,541	7,524	0,006%	0,002%	0,007%	0,001%
112	IGA2	48,934	8,611	0,083%	0,008%	0,055%	0,018%
113	IGHG1	49,328	8,048	3,535%	0,613%	2,443%	0,107%
114	IGHM	49,439	6,44	0,907%	0,070%	0,562%	0,025%
115	TUBB1	50,326	4,942	0,000%	0,000%	0,000%	0,000%
116	KRT16	51,267	4,848	0,029%	0,002%	0,011%	0,003%
117	CFP	51,276	7,697	0,001%	0,001%	0,000%	0,000%
118	FGG	51,511	5,323	1,971%	0,264%	2,117%	0,052%
119	KRT14	51,621	4,955	0,024%	0,004%	0,008%	0,001%
120	HPX	51,676	6,669	0,389%	0,024%	0,352%	0,004%
121	CPN1	52,286	7,022	0,002%	0,002%	0,003%	0,001%
122	CLU	52,494	5,973	0,106%	0,023%	0,121%	0,011%
123	SERPINC1	52,602	6,399	0,217%	0,026%	0,253%	0,029%
124	GC	52,917	5,302	0,355%	0,090%	0,511%	0,064%
125	SIGLEC16	52,991	8,98	0,087%	0,050%	0,138%	0,115%
126	AGT	53,154	6,003	0,027%	0,006%	0,038%	0,006%
127	LBP	53,383	6,387	0,005%	0,001%	0,008%	0,003%
128	A1BG	54,253	5,596	0,023%	0,004%	0,046%	0,001%
129	VTN	54,305	5,519	0,079%	0,002%	0,084%	0,013%
130	SERPINF2	54,565	5,986	0,018%	0,006%	0,022%	0,006%
131	SERPING1	55,154	6,232	0,106%	0,006%	0,079%	0,002%
132	FGB	55,928	8,11	1,343%	0,214%	1,511%	0,168%
133	IGHD	56,224	7,767	0,004%	0,001%	0,006%	0,001%
134	SERPIND1	57,07	6,58	0,022%	0,005%	0,031%	0,005%
135	KRT10	59,51	4,751	0,311%	0,027%	0,246%	0,025%
136	HRG	59,578	7,189	0,086%	0,029%	0,041%	0,007%
137	KRT6C;KRT6A	60,024	7,857	0,004%	0,001%	0,002%	0,001%
138	KRT6B	60,066	7,857	0,024%	0,003%	0,011%	0,001%
139	KRT9	62,129	5,02	0,248%	0,031%	0,162%	0,037%
140	PGLYRP2	62,216	7,262	0,003%	0,001%	0,003%	0,001%
141	KRT5	62,378	7,556	0,023%	0,004%	0,019%	0,002%
142	C9	63,173	5,34	0,010%	0,004%	0,016%	0,004%
143	IGM	63,485	8,187	0,001%	0,000%	0,001%	0,000%
144	KPRP	64,135	8,046	0,001%	0,001%	0,000%	0,001%
145	C8A	65,163	6,124	0,009%	0,001%	0,010%	0,001%
146	LGALS3BP	65,33	4,999	0,002%	0,000%	0,003%	0,000%
147	KRT2	65,432	6,582	0,101%	0,008%	0,091%	0,004%
148	CFI	65,75	7,281	0,011%	0,001%	0,010%	0,001%
149	IGFALS	66,034	6,455	0,011%	0,001%	0,003%	0,002%
150	KRT1	66,038	8,045	0,775%	0,061%	0,508%	0,033%

151	C4BPA	67,033	7,007	0,149%	0,015%	0,110%	0,003%
152	C8B	67,046	7,924	0,005%	0,001%	0,005%	0,002%
153	F12	67,791	7,533	0,016%	0,001%	0,011%	0,001%
154	AFM	69,068	5,627	0,006%	0,003%	0,013%	0,002%
155	ALB	69,366	5,974	32,617%	1,630%	37,754%	2,713%
156	F2	70,036	5,618	0,026%	0,007%	0,020%	0,005%
157	KLKB1	71,369	8,015	0,030%	0,006%	0,010%	0,004%
158	KNG1	71,957	6,475	0,165%	0,018%	0,134%	0,015%
159	PROS1	75,122	5,41	0,013%	0,004%	0,010%	0,003%
160	F13B	75,51	6,075	0,050%	0,023%	0,036%	0,009%
161	FERMT3	75,952	6,654	0,000%	0,001%	0,000%	0,000%
162	C1S	76,684	4,713	0,006%	0,001%	0,006%	0,003%
163	TF	77,063	6,795	3,044%	0,042%	3,234%	0,286%
164	C1R	80,118	5,894	0,002%	0,000%	0,003%	0,001%
165	PBXIP1	80,642	5,104	0,005%	0,001%	0,008%	0,001%
166	C2	83,267	7,129	0,012%	0,002%	0,015%	0,003%
167	PIGR	83,283	5,502	0,000%	0,000%	0,004%	0,001%
168	CFB	85,532	6,727	0,193%	0,008%	0,237%	0,031%
169	GSN	85,696	5,98	0,067%	0,003%	0,034%	0,006%
170	ITGB3	87,057	4,964	0,001%	0,000%	0,000%	0,000%
171	PLG	90,568	6,221	0,181%	0,032%	0,140%	0,027%
172	C7	93,517	6,146	0,021%	0,004%	0,020%	0,002%
173	FGA	94,972	5,733	2,487%	0,113%	2,624%	0,090%
174	ITIH1	101,39	6,466	0,012%	0,002%	0,014%	0,001%
175	ITIH4	103,36	6,667	0,163%	0,026%	0,178%	0,014%
176	C6	104,79	6,418	0,015%	0,001%	0,010%	0,001%
177	ITIH2	106,46	6,544	0,035%	0,008%	0,051%	0,009%
178	CP	122,2	5,443	0,107%	0,024%	0,175%	0,028%
179	STXBP5	127,57	6,969	0,000%	0,000%	0,004%	0,006%
180	THBS1	129,38	4,569	0,003%	0,000%	0,000%	0,000%
181	CFH	139,09	6,7915	0,137%	0,011%	0,128%	0,009%
182	ATAD2	158,55	6,02	0,001%	0,000%	0,001%	0,001%
183	A2M	163,29	6,149	0,294%	0,031%	0,445%	0,035%
184	ABCA10	175,79	6,33	0,000%	0,000%	0,000%	0,000%
185	C3	187,15	6,264	1,477%	0,092%	1,612%	0,080%
186	C5	188,3	6,21	0,017%	0,002%	0,023%	0,002%
187	C4B	192,75	6,949	0,000%	0,000%	0,000%	0,000%
188	C4A	192,78	6,747	0,260%	0,018%	0,238%	0,002%
189	MYH9	226,53	5,402	0,001%	0,000%	0,000%	0,000%
190	FN1	262,62	5,432	0,031%	0,007%	0,030%	0,002%
191	TLN1	269,76	5,805	0,002%	0,000%	0,001%	0,000%
192	FLNA	280,74	5,769	0,000%	0,000%	0,000%	0,000%
193	HRNR	282,39	10,139	0,012%	0,002%	0,005%	0,000%
194	CELSR2	317,45	5,039	0,002%	0,003%	0,002%	0,002%
195	DSP	331,77	6,501	0,000%	0,000%	0,000%	0,000%
196	FLG	435,16	9,354	0,000%	0,000%	0,000%	0,000%
197	LPA	501,31	5,61	0,000%	0,000%	0,000%	0,000%
198	APOB	515,6	6,729	0,070%	0,004%	0,118%	0,003%
199	RYR2	564,56	5,796	0,002%	0,001%	0,002%	0,001%
